# Supplementary material for: Modelling Skylarks (Alauda arvensis) to Predict Impacts of Changes in Land Management and Policy: Development and Testing of an Agent-Based Model
Source: PLoS One. 2013 Jun 6;8(6):e65803. doi: 10.1371/journal.pone.0065803 (PMC3675089; doi:10.1371/journal.pone.0065803)
Supplement: Supporting Information S4 — The skylark ODdox as a zipped archive. (ZIP) [file pone.0065803.s004.zip › Skylark_ODdox/class_movement_map-members.html]

ALMaSS Skylark ODdox: Member List


ALMaSS Skylark ODdox
 2.0


- Main Page
- Related Pages
- Classes
- Files

- Class List
- Class Index
- Class Hierarchy
- Class Members

MovementMap Member List

This is the complete list of members for MovementMap, including all inherited members.

|  |  |  |
| --- | --- | --- |
| GetMapValue(unsigned x, unsigned y) | MovementMap |  |
| Init(int spref) | MovementMap | protected |
| m\_ALandscape | MovementMap | protected |
| m\_TheMap | MovementMap |  |
| maxx | MovementMap |  |
| maxy | MovementMap |  |
| MovementMap(Landscape \*L, int spref) | MovementMap |  |
| SetMapValue(unsigned x, unsigned y, unsigned value) | MovementMap |  |
| SetMapValue0(unsigned x, unsigned y) | MovementMap |  |
| SetMapValue1(unsigned x, unsigned y) | MovementMap |  |
| SetMapValue2(unsigned x, unsigned y) | MovementMap |  |
| SetMapValue3(unsigned x, unsigned y) | MovementMap |  |
| ~MovementMap() | MovementMap |  |


- Generated on Thu Jan 10 2013 13:15:36 for ALMaSS Skylark ODdox by
   1.8.1.1
